# Retrograde transgastric jejunostomy for nutritional management and aspiration prevention in cases with severe malignant esophageal strictures

**DOI:** 10.1002/deo2.321

**Published:** 2023-11-20

**Authors:** Takeshi Yamashita, Koji Otsuka, Satoru Goto, Tomotake Ariyoshi, Kentaro Motegi, Masahiro Kohmoto, Akira Saito, Yoshihito Sato, Yutaka Kishimoto, Masahiko Murakami

**Affiliations:** ^1^ Esophageal Cancer Center Showa University Hospital Tokyo Japan

**Keywords:** aspiration pneumonia, esophageal cancer, nutrition, retrograde endoscopy, transgastric jejunostomy

## Abstract

Locally advanced esophageal cancer often presents with dysphagia and can be complicated by aspiration pneumonia. Therefore, nutritional management is important to prevent pneumonia. Enteral nutrition via gastrostomies is common in esophageal cancer patients. Here, we describe the efficacy of nutritional management using a gastrojejunostomy tube retrogradely inserted in the esophagus through gastrostomy to simultaneously drain accumulated fluid on the proximal side of a malignant stricture. We performed this procedure for two cases with severe malignant strictures using two types of endoscope insertion. A 57‐year‐old male patient (Case 1) underwent a retrograde insertion of a gastrojejunostomy tube for severe esophageal malignant stricture with severe nausea and salivary reflux. After a narrow endoscope was inserted through the gastrostomy fistula, a gastrojejunostomy tube was inserted alongside a guidewire allowing the patient to undergo definitive chemoradiotherapy without symptoms. An 82‐year‐old male patient (Case 2) was scheduled for a minimally invasive esophagectomy following neoadjuvant chemotherapy after gastrostomy. However, the patient developed aspiration pneumonia due to salivary reflux; before surgery, a narrow nasal endoscope was inserted and passed through the strictures. The percutaneous endoscopic transgastric jejunostomy catheter was retrogradely inserted alongside the guidewire. In patients with malignant strictures and salivary reflux, retrograde insertion of gastrojejunostomy tubes can simultaneously provide enteral nutrition and saliva drainage.

## INTRODUCTION

Patients with locally advanced esophageal cancer with severe strictures often experience dysphagia, making the prevention of aspiration pneumonia during surgical intervention necessary, along with preoperative nutritional management. Aspiration pneumonia can occur due to salivary reflux or fluid accumulation on the proximal side of the malignant esophageal stricture. Although drainage using a nasogastric tube is the most commonly employed treatment approach, performing an initial esophagectomy may be useful. Our institution is a high‐volume center for minimally invasive esophagectomies in Japan,^1^ and according to our practice guidelines for esophageal cancer treatment, locally advanced esophageal cancer is better treated with neoadjuvant therapy for R0 resection.^1^, ^3^ In this study, we delivered enteral nutrition by gastrojejunostomy tube insertion via gastrostomy with simultaneous retrograde drainage of fluid accumulation of the esophageal malignant stricture from the proximal side.

## CASE REPORTS

**FIGURE 1:**
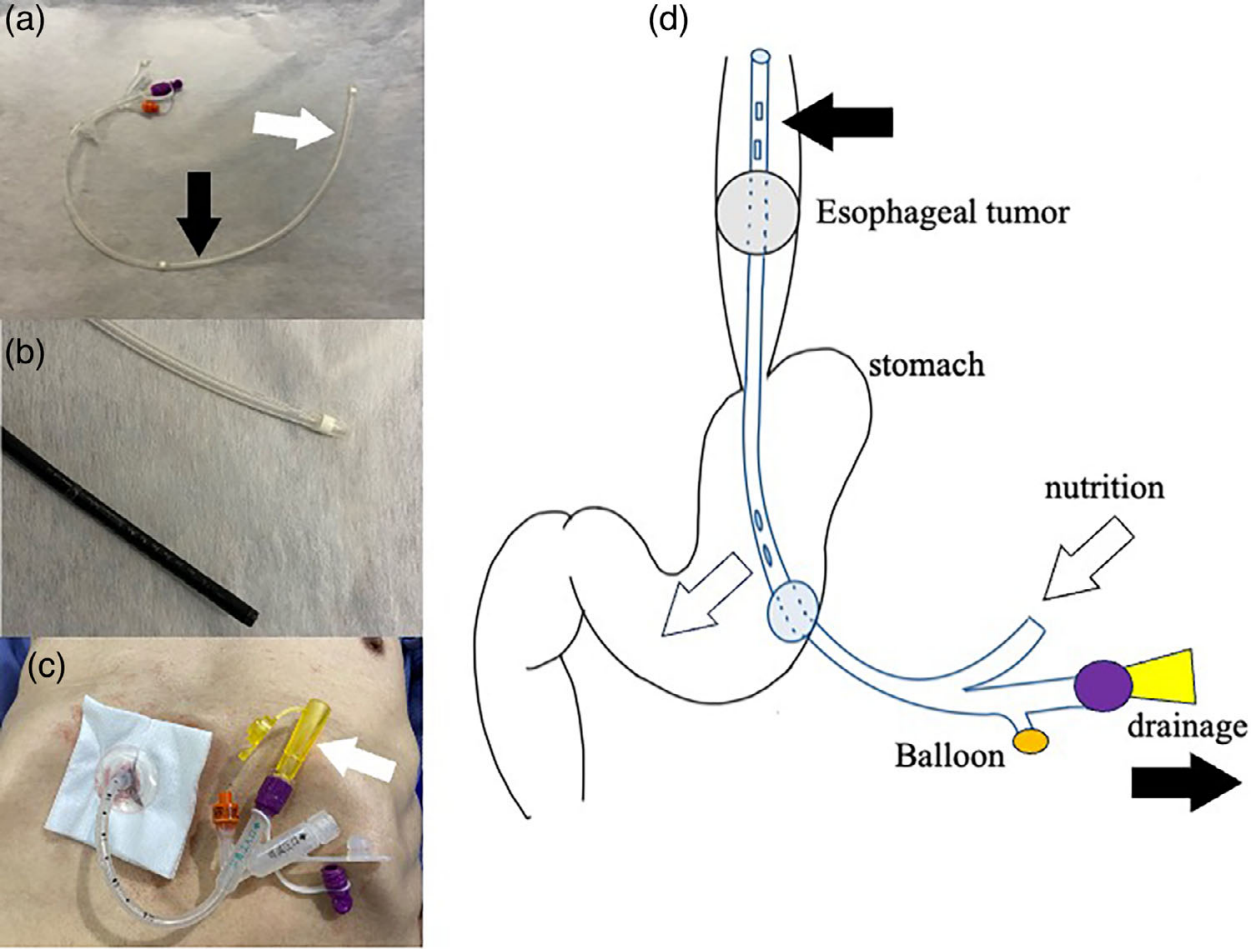
Gastrojejunostomy catheter. (a) *Cliny PEG‐J Catheter by Create Medic Co., Ltd*. There are holes for nutrition (white arrow) at the tip of the catheter and for drainage (black arrow) below the balloon. (b) 16Fr percutaneous endoscopic transgastric jejunostomy catheter is almost the same size as an ultrathin endoscope. (c) A yellow special connector (white arrow) is needed at the drainage side of the catheter. (d) An illustration of nutrition management (white arrow) and drainage (black arrow) using percutaneous endoscopic transgastric jejunostomy.

We typically use the Cliny percutaneous endoscopic transgastric jejunostomy (PEG‐J) catheter (Create Medic Co., Ltd.; Figure 1a), which has holes for nutrition and gastric juice drainage, at the catheter tip and below the balloon, respectively (Figure 1a), thus simultaneously allowing for both procedures. In particular, a 16‐Fr catheter was used because of its size compatibility with an ultrathin endoscope (GIF‐1200N; Olympus Co.; Figure 1b) and it can be inserted if the ultrathin endoscope can pass through a malignant stricture. Percutaneous endoscopic gastrostomy (PEG) was performed before the insertion of the catheter.

The nurses received a thorough explanation to avoid mistakes in tube management, as reverse connections were needed; the nutrition tube was connected to the drainage side and the special connector (Figure 1c). The nutritional management is illustrated in Figure 1d

### Case 1

**FIGURE 2:**
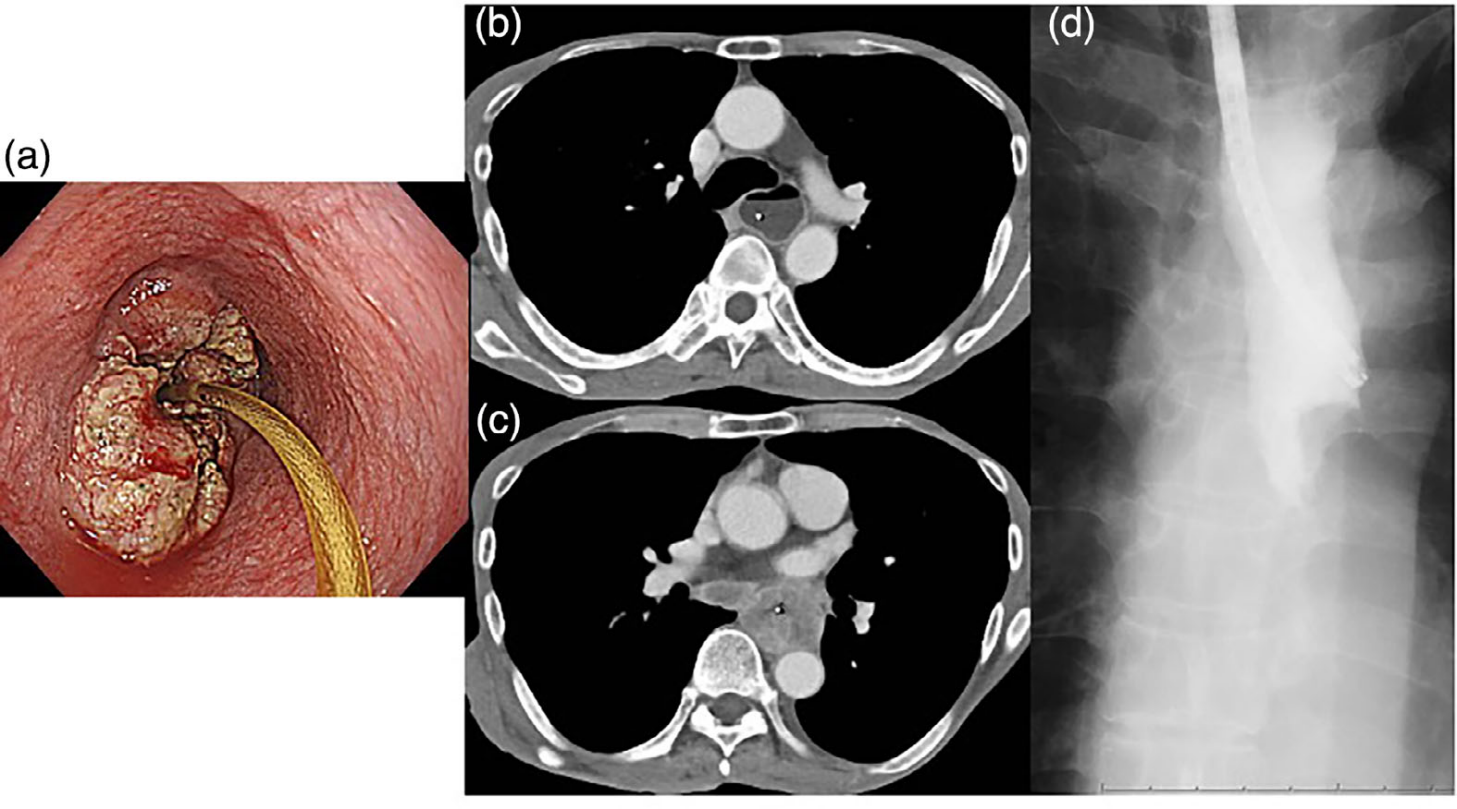
Endoscopy, computed tomography, and radiographic findings in Case 1. (a) Tumor has a severe stricture which the narrow nasogastric tube can pass through. (b) Computed tomography shows the dilatation of the esophagus at the proximal side of the malignant stricture. (c) Computed tomography shows the esophageal tumor with lymph node enlargement. (d) The ultrathin endoscope cannot pass through the stricture, and Gastrografin pooling can be observed.

**FIGURE 3:**
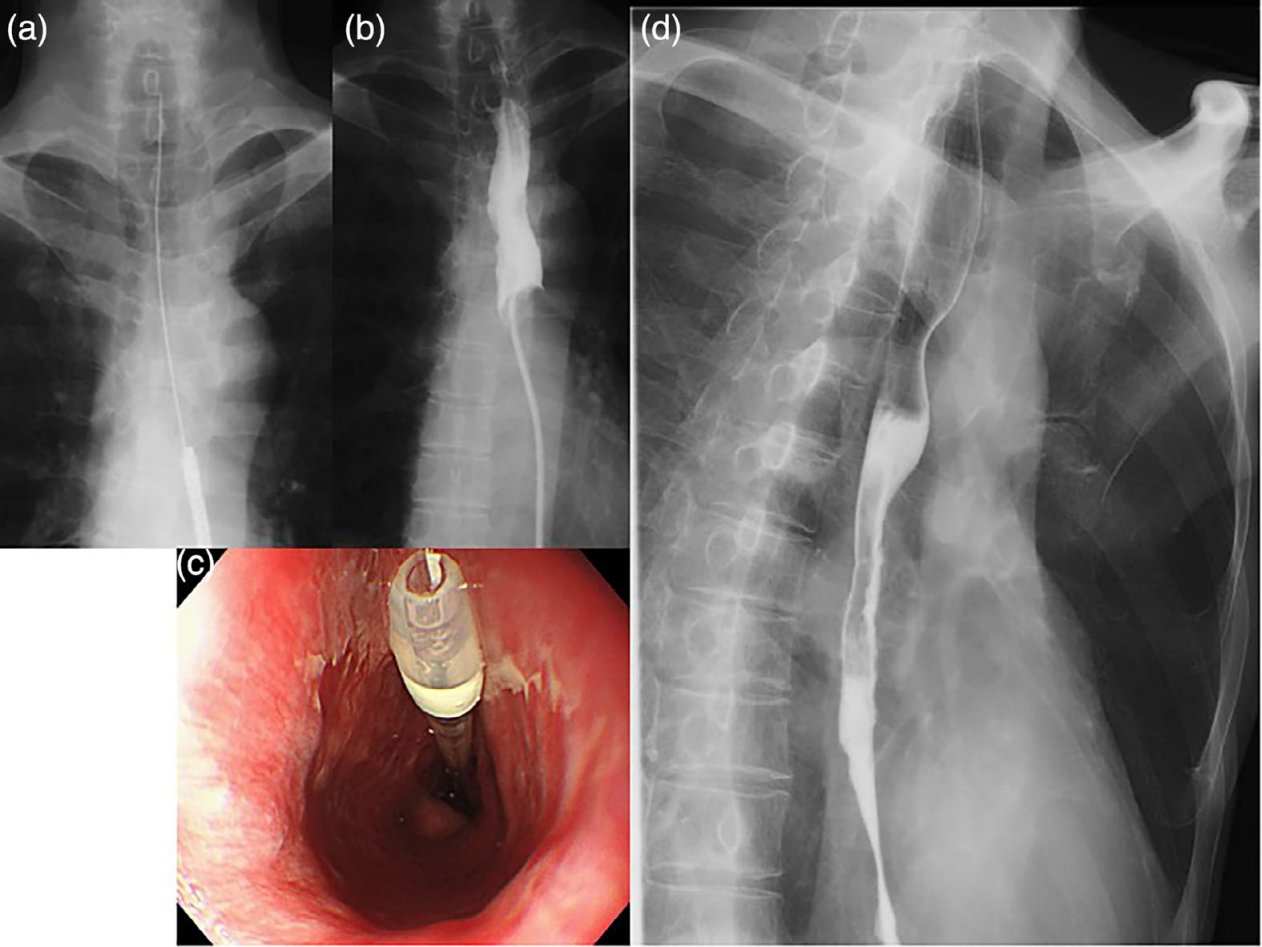
The insertion of percutaneous endoscopic transgastric jejunostomy retrogradely and the improvement of the stricture after definitive chemoradiotherapy in Case 1. (a,b) The ultrathin endoscope is inserted through the gastrostomy stoma to a point below the stricture, and a guidewire is passed through the stricture. (c) The tip of the percutaneous endoscopic transgastric jejunostomy catheter in the cervical esophagus on oral endoscopy is shown. (d) Contrast test after chemoradiotherapy shows the stricture improved and the pooling of Gastrografin disappeared.

A 57‐year‐old male patient presented with recurrent vomiting after his advanced esophageal squamous cell carcinoma diagnosis, which had invaded the left main bronchus and was accompanied by severe esophageal stricture and mediastinal lymph node metastases (Figure 2). Definitive chemoradiotherapy after PEG was planned; however, improving nausea and salivary reflux was difficult. Thus, we performed retrograde insertion of a PEG‐J catheter. As the guidewire could not pass through the malignant stricture by peroral, a narrow endoscope was inserted through the gastrostomy fistula after gastrostomy tube removal, and was followed by guidewire and PEG‐J catheter insertion (Figure 3a–c). Under nutritional management, the patient underwent definitive chemoradiotherapy with 5‐fluorouracil and cisplatin with 60‐Gy radiation. Four days post‐chemoradiotherapy, the patient resumed oral fluid intake and was discharged from the hospital (Figure 3d).

### Case 2

**FIGURE 4:**
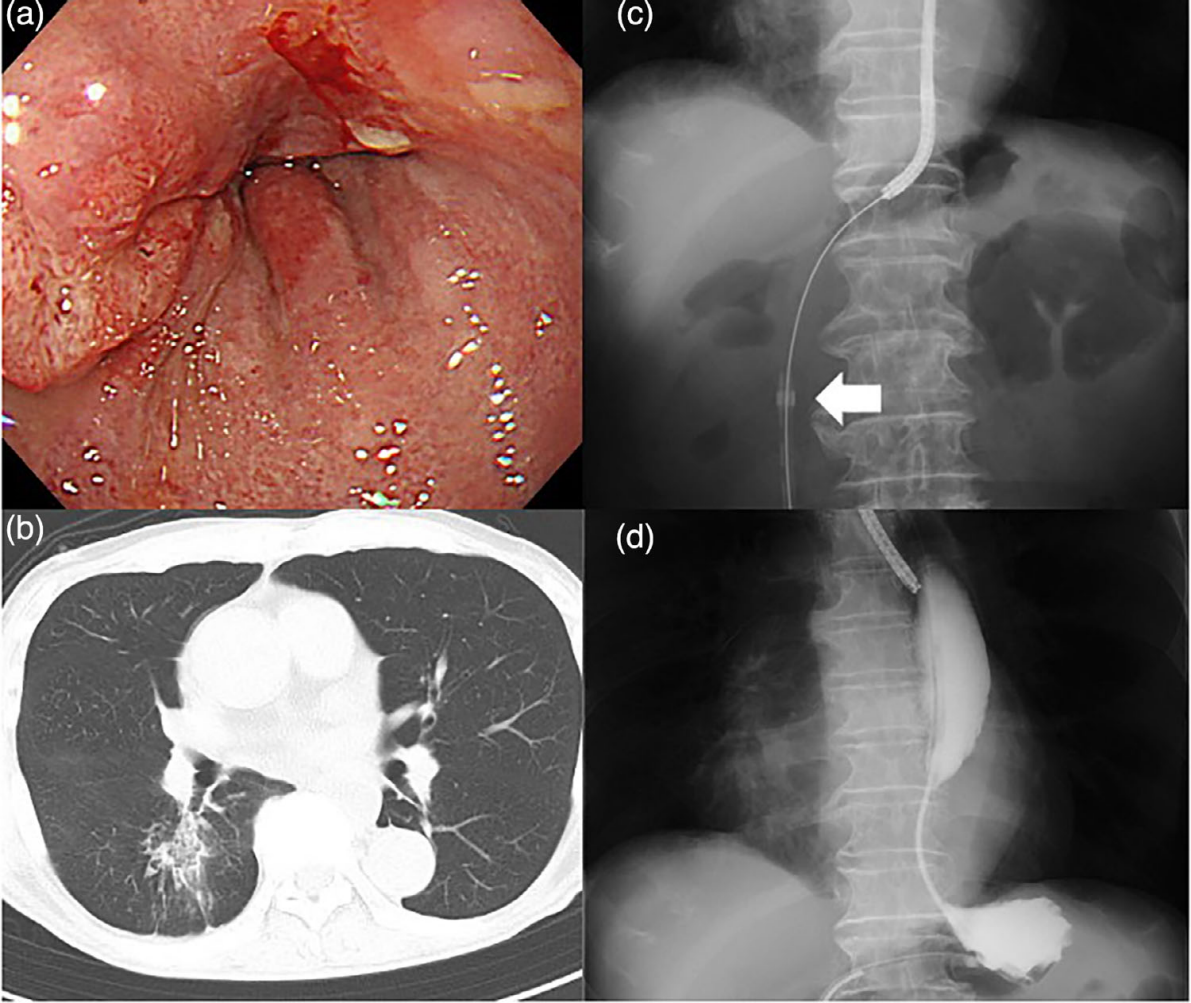
The insertion of percutaneous endoscopic transgastric jejunostomy retrogradely in Case 2. (a) Endoscopy and computed tomography findings of the tumor with the stricture. (b) The pooling of saliva caused aspiration pneumonia prior to management. (c) An ultrathin endoscope is passed through the stricture, and a percutaneous endoscopic transgastric jejunostomy catheter is inserted retrogradely from the gastrostomy stoma under the guidewire. The white arrow indicates the tip of the PEG‐J catheter. (d) Contrast test with Gastrografin shows the dilatation of the esophagus and the malignant stricture after catheter insertion.

An 82‐year‐old male patient presented with dysphagia and was diagnosed with esophageal squamous cell carcinoma with mediastinal and abdominal lymph node metastases and esophageal stricture (Figure 4a). We planned a minimally invasive esophagectomy following neoadjuvant chemotherapy after PEG; however, the esophageal cancer treatment was delayed because of coronavirus disease 2019. Although the tumor size was decreased by neoadjuvant chemotherapy, the stricture worsened, and the patient developed aspiration pneumonia due to salivary reflux (Figure 4b). We performed retrograde insertion of a PEG‐J catheter. The gastrostomy tube was removed after a narrow endoscope was inserted and passed through the stricture and gastrostomy fistula. The endoscope was then removed, and the PEG‐J catheter was retrogradely inserted along the guidewire (Figure 4c,d). As the symptoms resolved and pneumonia improved, the patient underwent a minimally invasive esophagectomy after physiotherapy. The patient ate a solid meal 8 days post‐surgery and was discharged 22 days after surgery.

## DISCUSSION

Patients who undergo esophageal resection have an increased risk of complications and malnutrition, thus requiring a perioperative nutritional assessment.^3^ Severe malignant strictures often lead to dysphagia, particularly for liquids, leading to saliva retention in the proximal esophagus. This sometimes causes vomiting and aspiration pneumonia, indicating enteral support, preferably using the gastrointestinal tract with selective use of feeding tubes.^3^ While a nasogastric tube is a less invasive method of enteral feeding, it carries long‐term risks of nonelective extubation and tube misplacement, blockage, reflux, and aspiration.^4^

Esophageal stent placement provides immediate dysphagia resolution, allowing patients to maintain oral nutrition, and has been shown to substantially improve dysphagia and allow oral nutrition during neoadjuvant therapy in patients with locally advanced esophageal cancer. However, it should not be routinely used as a bridge to surgery given its reported lower R0 resection rate, higher locoregional recurrence and postoperative complication rates, and shorter median time to recurrence and 3‐year overall survival.^5^

PEG is a viable enteral nutritional route for patients with upper gastrointestinal tract and head and neck malignancies, as palliation in inoperable cases, or prior to radiotherapy or chemotherapy.^6^ Preoperative PEGs for esophageal cancer are safe and do not compromise future anastomoses.^7^

PEG‐J involves gastrostomy with a jejunal extension tube, through which the feeding solution can be administered, and gastric decompression functions are performed.^6^ PEG‐Js can prevent aspiration pneumonia arising from gastroesophageal reflux of the gastric feeds.^8^ PEG‐J, which uses jejunal extension tubes placed through PEG tubes, carries the risk of tube blockage and limited gastric decompression because of the tube size through the PEG. However, these limitations are avoided with the Cliny PEG‐J Catheter as the tube size is larger than that of a jejunal extension tube^8^; furthermore, its design ensures that there is minimal risk of dislocation or deviation towards the anal side due to its extended length, with the tip placed at the cervical esophagus, making it more convenient to use than other PEG‐J catheters.

We performed PEGs for nutritional management; however, symptoms such as dysphagia, nausea, and salivary reflux could not be resolved. As the symptoms improved, we planned a retrograde endoscopic treatment. Retrograde procedures using gastrostomies are sometimes effective in cases where the transoral approach is difficult. Some previous reports have described unique endoscopic treatments, including rendezvous endoscopy to perform tissue puncture, dilatation, and stenting for esophageal strictures.^9^ Our procedure is less invasive than esophageal stenting because it can be completed provided an ultrathin endoscope (outer diameter, 5.4 mm) can pass through the stricture of the tumor. There are some limitations to this procedure. It may not be suitable for patients with cervical esophageal cancer because of the catheter length. Additionally, in severe strictures in which even a guidewire cannot pass through, its insertion may be difficult. However, nutritional injection management should be considered because of the reverse use between the injection side of the nutritional liquid and the drainage side. If the liquid is administered from the drainage side (usually the injection side), aspiration pneumonia may occur at a high rate.

We found no English‐language reports of any similar procedures in PubMed up to May 2023 using the keywords “retrograde,” “PEG‐J,” and “esophageal cancer”; however, we found a report on the same management approach written in Japanese,^10^ but while it presented the efficacy of this procedure using a PEG‐J it lacked sufficient tube insertion procedure details.

The PEG‐J catheter is more convenient and useful than narrow tubes for gastrostomy stomas. In our study, mistakes in the management of tubes were avoided in both patients. We believe that retrograde insertion of the PEG‐J catheter for malignant esophageal strictures is an easily performed, safe, and less invasive approach for nutritional management combined with saliva drainage from the proximal side of the esophageal strictures during oncologic treatments. Therefore, this procedure can be used as a bridge to surgery.

Nutritional management is important during esophageal cancer treatment. In patients with malignant strictures and salivary reflux, retrograde insertion of a PEG‐J catheter can simultaneously provide enteral nutrition and saliva drainage. In the future, it would be beneficial to compare this procedure with other treatments such as esophageal stents for the prevention of aspiration pneumonia in clinical trials.

## CONFLICT OF INTEREST STATEMENT

None.

## ETHICAL STATEMENT

This study was approved by the Evaluation Committee for Highly Difficult New Medical Technologies and Unapproved New Pharmaceuticals at Showa University Hospital. Informed consent was obtained from both patients.
